# “I’m not feeling alone in my experiences”: How newly diagnosed autistic adults engage with a neurodiversity-affirming “Welcome Pack”

**DOI:** 10.1177/13623613251335070

**Published:** 2025-04-25

**Authors:** Chris Edwards, Abigail MA Love, Ru Ying Cai, Melanie Heyworth, Alexandra Johnston, Fiona Aldridge, Vicki Gibbs

**Affiliations:** 1 Autism Spectrum Australia (Aspect), Australia; 2 Griffith University, Australia; 3 The University of Melbourne, Australia; 4 La Trobe University, Australia; 5 Reframing Autism, Australia; 6 Macquarie University, Australia; 7 The University of Sydney, Australia

**Keywords:** autistic adult, diagnosis, late-diagnosed, neurodiversity-affirming, post-diagnostic, self-guided

## Abstract

Receiving an autism diagnosis in adulthood can be profoundly transformative, yet many newly diagnosed autistic adults experience a lack of accessible, affirming post-diagnostic support. This study explores how a self-guided, neurodiversity-affirming resource—the “Welcome Pack”—supports newly diagnosed autistic adults in navigating their evolving identity. Using a qualitative approach, we recruited 11 autistic adults without intellectual disability in Australia who had received a diagnosis within the past 6 months. Participants engaged with the “Welcome Pack” over a 2-month period before taking part in semi-structured interviews. Reflexive thematic analysis generated four key themes: (1) a sense of belonging through community and representation, as participants found validation in shared narratives; (2) the “Welcome Pack” as one of many tools supporting their self-understanding; (3) emotional reflections, including both relief and grief over years of masking; and (4) challenges in self-advocacy and educating others. While participants described the “Welcome Pack” as an important and validating resource, they also highlighted the need for additional structured peer support and practical guidance. These findings underscore the value of integrating self-guided resources within broader post-diagnostic ecosystems, ensuring that newly diagnosed autistic adults receive timely, accessible, and neurodiversity-affirming support as they navigate their identity.

**Lay Abstract:**

## Abstract (plain language summary)

Many autistic people receive a diagnosis in adulthood, often after years of feeling different but not knowing why. While a diagnosis can bring relief, it can also be overwhelming, with few accessible and affirming post-diagnostic supports available. This study explores how a free, self-guided resource—the “Welcome Pack”—helps newly diagnosed autistic adults navigate this transition. The “Welcome Pack,” developed by autistic people, includes personal stories, practical advice, and reflection activities to support self-understanding and identity development. We worked with 11 autistic adults in Australia who had been diagnosed within the past 6 months. After using the “Welcome Pack” for 2 months, they shared their experiences through interviews. Our analysis identified four key themes. First, participants felt validated and less alone when reading personal stories from other autistic people. Second, they found the “Welcome Pack” useful but emphasized the need for additional resources. Third, they reflected on the emotional impact of their diagnosis, describing both relief and grief over years of masking (hiding or suppressing their autistic traits to fit in or avoid judgment). Finally, they faced challenges in self-advocacy and explaining autism to others. Overall, participants valued the “Welcome Pack” as a helpful starting point, but they also highlighted the need for more structured peer support and workplace advocacy tools. These findings suggest that while self-guided resources can provide meaningful support, they should be integrated with broader community and professional services to better meet the needs of newly diagnosed autistic adults.

The pathway to receiving an autism diagnosis in adulthood is often complex, emotional, and deeply personal. For many, this journey begins with a pivotal life event—such as the diagnosis of a child or a personal crisis—that prompts self-reflection and a search for answers (Cage et al., 2024; Crane et al., 2018; de Broize et al., 2022; Nayyar et al., 2025). For others, the process unfolds more gradually, as a lifetime of feeling “different” converges with increased awareness of autism in adulthood (de Broize et al., 2022; Gellini & Marczak, 2023; Huang et al., 2020; Kelly et al., 2024; Kiehl et al., 2024).

Regardless of the starting point, the diagnostic process is rarely straightforward. Many adults face significant barriers to diagnosis, including lengthy waiting lists, limited access to knowledgeable professionals, systemic biases that disproportionately impact women and other marginalized groups, and the overshadowing of autism traits by co-occurring mental health concerns (Cook et al., 2024; Crane et al., 2018; de Broize et al., 2022; Huang et al., 2020; Kelly et al., 2024; Kiehl et al., 2024; Leedham et al., 2020; Nayyar et al., 2025; Wilson et al., 2023). Masking or camouflaging autistic traits further complicates the process, as individuals may consciously or unconsciously suppress traits to meet societal expectations, delaying recognition and diagnosis (Kelly et al., 2024; Kiehl et al., 2024; Wilson et al., 2023). Furthermore, camouflaging often persists even after diagnosis and is associated with increased mental health difficulties (Cook et al., 2021). This issue is particularly pronounced in autistic people who identify as women, transgender, and/or non-binary, as they have been shown to camouflage more frequently and extensively than autistic people who identify as cisgender men (Cook et al., 2021; Hull et al., 2020; McQuaid et al., 2022).

Receiving a formal diagnosis can evoke a wide range of emotions, from profound relief and validation to confusion, grief, or anger (Crane et al., 2018; Crompton et al., 2022; Gellini & Marczak, 2023; Huang et al., 2020; Kelly et al., 2024; Kiehl et al., 2024; Nayyar et al., 2025; Wilson et al., 2023). For many, however, the diagnosis serves as a powerful framework to reinterpret past experiences, which can help foster compassion, self-acceptance, and provide a sense of clarity (de Broize et al., 2022; Gellini & Marczak, 2023; Kelly et al., 2024; Kiehl et al., 2024; Leedham et al., 2020; Wilson et al., 2023). While the period following an autism diagnosis in adulthood represents a critical time for support, many autistic adults report significant unmet needs during this transition, such as a lack of practical post-diagnostic resources, few opportunities for local peer connections, invalidation of their lived experiences, and little assistance in understanding and developing a positive autistic identity (Cage et al., 2024; Crane et al., 2018; Crompton et al., 2022; de Broize et al., 2022; Huang et al., 2020; Nayyar et al., 2025; Norris et al., 2024).

As awareness of the autism mental health crisis grows, it has become increasingly evident that receiving an autism diagnosis without post-diagnostic support is insufficient. While diagnosis can provide a framework for self-understanding, it often highlights pre-existing mental health challenges or introduces new difficulties, underscoring the need for comprehensive post-diagnostic support to help adults process their diagnosis and navigate their evolving identity (Mandy, 2022). Many autistic adults express a strong desire for opportunities to connect with like-minded individuals, support to process the emotional impact of their diagnosis, and access to practical, affirming information tailored to their needs, such as navigating educational and workplace adjustments (Cage et al., 2024; Crane et al., 2018; Crompton et al., 2022; Crowson et al., 2024; Norris et al., 2024). Although providers recognize the importance of post-diagnostic support, many face significant resourcing constraints that limit their ability to provide adequate care (Crane et al., 2018; Crompton et al., 2022). As a result, autistic adults are often left to navigate the vast and varied landscape of autism information independently—a process they find problematic due to the prevalence of outdated, pathologizing content and the lack of neurodiversity-affirming perspectives (Beresford & Mukherjee, 2025).

Despite the evident need for post-diagnostic support for autistic adults, research on such programs remains limited. One notable example is the United Kingdom-based program *Exploring Being Autistic*, a 10-week autistic-led, group-based program combining psychoeducation, practical support, and opportunities for peer connection. Evaluations of both face-to-face and online delivery models highlighted its promise, with participants reporting an increased sense of community and a more positive and practical outlook on being autistic (Crane et al., 2020, 2023). Similarly, evaluations of post-diagnostic services in the United Kingdom, including a 7-week multidisciplinary group program for newly diagnosed adults (Hatton & Lee, 2022) and Specialist Autism Teams providing extended post-diagnostic support (Beresford et al., 2020), emphasize the value of structured interventions in fostering understanding, self-acceptance, and community connection. In addition, *Understanding You, Discovering You* (Cooper et al., 2024; Davies et al., 2024), demonstrated how a codesigned online peer support program for autistic young adults fosters self-discovery, social connection, and practical skill development.

While these programs can be beneficial, their reliance on skilled facilitators and the associated financial, logistical, and geographical barriers (Crowson et al., 2024) can limit accessibility and sustainability. With increasing numbers of autistic adults seeking support, there is a clear need for cost-effective and scalable post-diagnostic options (Mandy, 2022). Self-guided interventions tailored for autistic adults have emerged as a promising complement to facilitated programs, offering accessible support in areas such as self-compassion (Cai et al., 2024; Edwards et al., 2024) and disclosure decision-making (Han et al., 2024).

## “Welcome Pack”: a neurodiversity-affirming resource

Building on the potential of self-guided resources, Reframing Autism, an Australian autistic-led not-for-profit organization, developed the “Welcome Pack”—a free, neurodiversity-affirming, self-guided resource designed for adults who have recently discovered or identified as autistic, whether through formal diagnosis or self-identification. Unlike existing programs that often require facilitation, the “Welcome Pack,” available in digital and downloadable formats, is designed to be self-directed, addressing barriers such as cost, accessibility, and geographical limitations. While the “Welcome Pack” aims to fill unmet needs in post-diagnostic care, little is known about how newly diagnosed autistic adults experience such self-guided tools and how these tools support their post-diagnostic journey. To address this gap, this study explores the following research question: How do newly diagnosed autistic adults experience the “Welcome Pack” as a self-guided resource, and how does it contribute to their broader post-diagnosis journey?

## Method

### Positionality

This research represents a meaningful partnership between autistic researchers and autism organizations. The Aspect Research Centre for Autism Practice (ARCAP), which includes both autistic and non-autistic autism researchers within a national Australian not-for-profit organization, led the research component of the study. The lead researcher (C.E.) identifies as a late-diagnosed autistic adult, and two additional autistic autism researchers from Reframing Autism (A.J. and M.H.), both diagnosed in adulthood, played key roles. These researchers (A.J. and M.H.) also previously contributed to the development of the “Welcome Pack.” Their own post-diagnostic support did not adequately meet their needs, reinforcing the gaps in existing services.

In addition, the team included diagnosticians (V.G. and F.A.) who had experience providing adult autism assessments but were limited in their resourcing to offer comprehensive post-diagnostic support. While the research was initially connected to a potential funding opportunity, the team recognized its importance and pursued it even without external funding. This statement reflects both the positionality of the research team and the central role of community involvement in the study’s design and execution.

### “Welcome Pack”

“Welcome to the Autistic Community: A Welcome Pack” (“Welcome Pack”) was developed by Reframing Autism’s Intersectional Advisory Committee (A.J. was the co-design lead) and made freely available on the Reframing Autism website in March 2024 (https://reframingautism.org.au/service/welcome-pack/). The committee, comprised of 10 representatives from underrepresented and multiply marginalized groups within the autistic community, codesigned this resource to support adults who have recently received an autism diagnosis or have come to the realization that they are autistic. This research did not involve the development of the “Welcome Pack” itself; the resource was independently created by Reframing Autism prior to the study. Our study was an initial exploration of how newly diagnosed autistic adults engage with the “Welcome Pack,” aligning with the development phase of complex intervention research (Skivington et al., 2021).

The “Welcome Pack” provides a comprehensive guide that includes personal stories, insights, and practical strategies to foster self-understanding, self-compassion, and the development of a positive autistic identity. The resource is organized into seven key elements, framed by an introduction to Reframing Autism at the beginning, how it was created, and supplementary resources and contributor biographies at the end: (1) Discovering you are autistic; (2) Autism acceptance, self-compassion, and self-care; (3) Developing a positive autistic identity; (4) Reducing masking and camouflaging; (5) Living a good autistic life; (6) What we wish we had known; and (7) What we want others to know. The “Welcome Pack” adopts a neurodiversity-affirming perspective, emphasizing that autism is a natural variation of human diversity rather than a disorder to be “fixed.” The pack is designed to be user-friendly, allowing individuals to engage with its content in ways that suit their unique journey, whether by reading it in its entirety or exploring sections most relevant to them. In addition, it includes reflection prompts that encourage readers to consider their own experiences, helping them to better understand and accept their autistic identity. The pack is available in two formats: a PDF version (194 pages) that includes images and colors, and a Word version (102 pages) that omits these elements for ease of access.

### Procedure

We obtained ethical approval from the Griffith University Human Research Ethics Committee (2023/671). Initially, we provided promotional material to services conducting adult autism assessments in Australia through the research team’s own networks, enabling diagnosticians to pass on study information to their adult clients. Due to limited recruitment through diagnosticians, we adapted our approach to recruit publicly through social media and recruited participants from May 2024 to August 2024. Potential participants completed a suitability survey, and individuals were deemed eligible if they were aged 18 years or older, lived in Australia, and had received a formal autism diagnosis within the past 6 months. This study specifically focused on autistic adults without intellectual disability, addressing the significant gap in evidence regarding post-diagnostic support tailored to this group (Norris et al., 2024). The “Welcome Pack” is intended for anyone realizing they are autistic—whether formally diagnosed or self-identified—however, participation in this study was limited to those with a formal diagnosis. While we recognize and value self-identification as autistic, this research was designed to examine experiences tied to receiving a formal diagnosis, which shaped the eligibility criteria.

As part of our strategy to avoid imposter participants, we asked potential participants to provide an image of their formal diagnostic report as part of the suitability survey (Pellicano et al., 2024). To increase feelings of safety, only the lead researcher, who disclosed his autistic identity to participants, handled this documentation. From the initial pool of eligible participants (*n* = 38), only 14 provided a screenshot of their diagnostic report, meeting the verification criteria. At the end of the suitability survey, we directed all eligible participants to Reframing Autism’s website, where they could download and access the “Welcome Pack,” which was already available to the public for free. Participants also had the option to enter their mailing address if they preferred a physical copy of the Word version, free of charge. After having the “Welcome Pack” for approximately 2 months, we approached the 14 verified participants to invite them to an interview, allowing time for them to access the materials and process initial emotions. Of these, 11 agreed to participate in an interview.

### Participants

Table 1 summarizes participant demographic information. Six participants worked full-time, three were employed part-time or casually, one was unemployed but actively seeking work, and one was unemployed but not seeking work. Several participants reported co-occurring conditions: attention-deficit/hyperactivity disorder (ADHD, *n* = 5), anxiety (*n* = 3), and depression (*n* = 3). The following conditions were reported by one participant each, premenstrual dysphoric disorder (PMDD), complex post-traumatic stress disorder (C-PTSD), Irlen Syndrome, body dysmorphic disorder, and twice-exceptional (2e). Three participants reported no co-occurring conditions.

**Table 1.** Participant demographic information (*N* = 11).

Pseudonym	Age	Gender	Relationship	Ethnicity
Katie	39	Non-binary	Single	White
Robyn	39	Female	Married	White
Sam	46	Male	Married	Mixed
Liz	39	Female	Single	White
Nyla	23	Female	In a relationship (not de facto)	South East Asian
Peta	39	Female	Married	White
Bonnie	37	Female	Married	White
Mika	45	Female	Married	White
Tyler	39	Male	Single	Middle Eastern/White
Hayley	59	Female	Single	White
Sally	60	Female	In a de facto relationship	White

Participant names are pseudonyms assigned during data analysis.

### Interview

We offered participants the choice to engage in interviews via phone, video call, or written response, enabling them to select their most comfortable communication method. Four participants spoke with us by phone, five connected through video calls, and two shared their experiences in writing. We recorded each verbal interview and used Otter.ai for initial transcription, with the lead researcher carefully reviewing and refining each transcript while listening to the audio recording to ensure accuracy. Our conversations lasted between 24 and 63 min, averaging 36 min. We provided each participant with a $50 (AUD) gift card to acknowledge their valuable contribution to the research.

Our interviews were designed to explore participants’ post-diagnostic experiences and their engagement with the “Welcome Pack” rather than evaluate its effectiveness as an intervention. To ensure an open and participant-led discussion, we began with broad questions about how their autism diagnosis had influenced their self-understanding and daily life, including the types of support and information they sought during this period. We then invited participants to share their experiences with the “Welcome Pack,” including their initial impressions, any connections between its perspectives on autism and their own experiences, and how they engaged with its content. Discussions of the “Welcome Pack’s” seven main sections were guided by participants’ interests and reflections, allowing them to highlight particularly meaningful topics or identify areas they felt could be expanded or improved. The interviews concluded with an opportunity for participants to share any additional thoughts or reflections, ensuring a comprehensive understanding of their experiences. The interview schedule is included with the supplemental material.

### Data analysis

We used reflexive thematic analysis (RTA) as it allowed us to explore patterns in participants’ experiences with the “Welcome Pack” and their broader post-diagnostic journeys. RTA (Braun & Clarke, 2006; Braun et al., 2019) supported our constructionist epistemology, emphasizing the co-construction of meaning through both frequency and depth of themes in relation to our research question. We took an experiential orientation, focusing on participants’ personal reflections rather than broader societal constructions of autism.

The lead author, a late-diagnosed autistic adult, completed the coding process, ensuring that an autistic perspective was central to the initial stages of analysis. To enhance reflexivity, the three autistic researchers on the team collaboratively reviewed and refined preliminary themes before engaging the wider research team. This process included peer debriefing and discussion with non-autistic diagnosticians, allowing for critical reflection on how different positionalities shaped the interpretation of findings. Using a combination of semantic and latent coding, we examined both surface meanings and deeper assumptions, guided by an inductive approach. The analytical process was iterative, with ongoing discussions ensuring that the final themes were grounded in participant experiences while acknowledging the influence of researcher perspectives.

## Results

**Figure 1.**
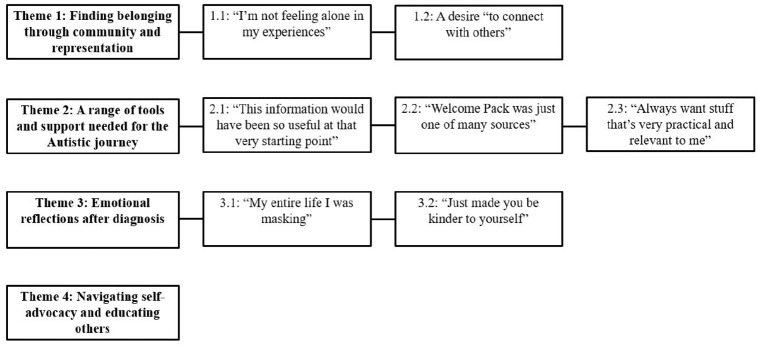
Thematic map of autistic adults’ experiences with the Welcome Pack and their post-diagnostic journey.

Our data analysis led to the generation of four themes and seven subthemes as shown in Figure 1.

### Theme 1: finding belonging through community and representation

The first theme created from participants’ accounts centers on the impact of representation and community in the early post-diagnostic period. This theme captures both the immediate resonance participants found within the “Welcome Pack” narratives and their subsequent desire for deeper community connections.

#### Subtheme 1.1: “I’m not feeling alone in my experiences.”

The biographical narratives within the “Welcome Pack” emerged as a particularly powerful element in participants’ early post-diagnostic experiences. These stories appeared to serve multiple functions: validation, recognition, and the creation of a sense of shared experience. As Liz noted, the first thing they went to was the “biographies . . . you want to know the people telling the story,” highlighting the immediacy with which newly diagnosed adults seek connection through others’ experiences.

Participants also emphasized how the diversity of narratives enhanced rather than diminished their sense of connection. Bonnie articulated this clearly, “when I looked at the welcome pack, and I was reading stories, and the spectrum of people that are in the stories, I think, are what make it really amazing.” This appreciation for diversity while maintaining connectedness emerged as a crucial aspect of “feeling part of a community” (Tyler). The transformative nature of this representation was particularly evident in Bonnie’s account:

Look at all these people that are out there doing this and loving this and seeing this and how they felt really similar to mine, and to be seen and heard is such a big value of mine, but never really happened in a way that did when I read that book [“Welcome Pack”]—was huge for me.

This sense of recognition resonated throughout participants’ accounts, with many describing how the stories offered validation and connection, such as “affirming I’m not alone” (Sally). These shared narratives enabled participants to reframe their past experiences through a new lens of understanding. As Bonnie reflected, they revealed “how I wasn’t the only one that treated myself kind of badly, and how there are these people out there that just understand you, which I felt like I wasn’t understood for my whole life.”

#### Subtheme 1.2: A desire “to connect with others.”

While the representational aspects of the “Welcome Pack” provided crucial initial validation, many participants expressed a need for more direct community engagement. This desire for tangible connections with other late-diagnosed autistic adults suggests that written narratives, while valuable, represent just one aspect of the post-diagnostic support needed. Mika articulated this gap clearly, “I guess that’s kind of the part that I haven’t really found yet, like, aside from my work colleague, you know, I would like to connect with others that are late diagnosed.” Notably, participants offered specific suggestions for structured community engagement. Nyla proposed,

I feel like if it was like a social group that we all kind of went through it (“Welcome Pack”) together. Like, each week we went through this section and this section, I feel like I would have made it fun.

The emphasis on positive, intentional community spaces was particularly striking. As Sam expressed,

I’d like to join a (peer) group that’s positive about that and like, that’s something that I really want to be involved in. I think that would be a good way of spending time with other people who’ve been diagnosed.

### Theme 2: a range of tools and support needed for the autistic journey

The second theme illuminates how newly diagnosed autistic adults engage with a variety of resources, including the “Welcome Pack,” as they navigate their post-diagnostic journey.

#### Subtheme 2.1: “this information would have been so useful at that very starting point.”

A consistent reflection across participants’ accounts was the recognition that having access to the “Welcome Pack” would have been most valuable during the initial stage of self-discovery, when they first began questioning whether they might be autistic, before receiving a formal diagnosis:

If I had this (“Welcome Pack”) right at the beginning, probably would have got even more out of it. So, think that, like the journey is, doesn’t really start at diagnosis. No, I think for most adults, if you’re getting into that point, you’ve probably done a lot of this exploration beforehand. (Robyn)

This was particularly relevant in fostering a sense of community through stories. As Nyla reflected, “at the start of me thinking that I might be autistic, I would have really loved those type of stories and just like knowing that my experience is valid.” In addition, some participants highlighted the challenges of searching for information about autism online, “really difficult to navigate . . . the challenge of information that’s not neuroaffirming . . . really outdated scientific information as well” (Robyn). As Peta explained, “it could have shaped my understanding so much [having the ‘Welcome Pack’ earlier], that I would have known more like, what to read or what not to read, what to believe.”

#### Subtheme 2.2: “Welcome Pack was just one of many sources.”

Participants frequently described engaging with multiple sources of information to better understand autism, with the “Welcome Pack” playing a complementary role within this broader ecosystem of support. Mika described how they were “doing so much research on all, you know, different websites and social platforms.” Personal connections were highlighted as particularly significant, as Liz shared, “because I have other resources . . . I have a few friends who work in the same sort of area as me, who are late diagnosed female Autistic. So that was my kind of go-to point.” This approach of collecting information from different sources was echoed by Mika who explained:

It’s kind of the cumulative effect of, you know, the “Welcome Pack” and the online communities. And I also have a work colleague who was late diagnosed as well . . . so I guess it was a combination of all those things.

Some participants actively sought out resources tailored to their specific circumstances. For example, Sally shared, “the most helpful [resource] being a podcast, The Late Discovered Club which specifically related to me as an older woman being diagnosed late in life.” Overall, there was a strong appreciation for how the “Welcome Pack” helped consolidate the information participants were already gathering. Mika remarked how “it [‘Welcome Pack’] kind of tied everything in nicely together.”

#### Subtheme 2.3: “Always want stuff that’s very practical and relevant to me.”

Participants reflected on the kinds of practical guidance they found useful and where they felt additional support was needed. Some described areas where the “Welcome Pack” could be expanded, while others spoke more broadly about the kinds of structured, hands-on strategies they needed to navigate life post-diagnosis. These areas were seen as critical during the transition into post-diagnosis life. As Bonnie explained,

People who are late diagnosed—we’ve gotten to where we are now, but I don’t know how many of us have healthy habits from all the masking we’ve done to get to this point. To come out the other side—Autistic, happy, and our actual Autistic selves—I feel like that’s when you need the support.

Masking and camouflaging emerged as key areas where participants sought deeper insights. Mika highlighted this need, stating:

I guess that masking and camouflaging bit, because that’s the area that I feel I need to work on the most. So that probably stood out the most. I probably could have read more on that. If you have more in there, I would have definitely dived a bit deeper there.

Similarly, Peta emphasized the importance of actional advice, expressing a desire for “the practical, tangible strategies of how you can actually embrace autism.” Practical advice for workplace advocacy and navigating support systems was also identified as an area requiring more depth. Robyn noted, “I think some of the topics were . . . little . . . I don’t know what the word is here, vague . . . The thing that I really wanted was this idea of, like, I guess, advocating and navigating the workplace.”

### Theme 3: emotional reflections after diagnosis

Engaging with resources like the “Welcome Pack” appeared to support participants in processing their diagnosis and reflecting on their emotions. As Bonnie shared, “I left reading this feeling good about myself for the first time, and that was really helpful.” Participants described complex emotional responses following their diagnosis, including grief over years of masking and self-misunderstanding, alongside newfound self-compassion and relief in better understanding themselves.

#### Subtheme 3.1: “my entire life I was masking.”

Participants reflected on the profound emotional toll of masking, often expressing grief and regret for the years spent concealing their true selves in order to fit in. As Bonnie shared,

Grief . . . but not because of the diagnosis, but because of how badly I treated myself for so many years . . . I pretty much beat myself, I’d say, for 35 years . . . grieving for not knowing how my brain works, for thinking that I was never as good as everyone else.

Masking was often described as a survival mechanism, but one that came at a significant psychological cost, “I was lucky that I got in my job. I got the economic benefits from it, but at the same time the psychological damage, I just call it damage, is quite profound, because you had to push through it” (Hayley). Several participants acknowledged how masking became second nature, making it difficult to discern between their authentic selves and their camouflaged behaviors:

My entire life I was masking . . . I didn’t even realize I was doing it. I just did it because, you know, I’d been conditioned to go that way . . . the more I think about it and I’m aware of it, the more I’m like, well, how do I stop doing that when I can? (Liz)

Despite these challenges, some participants described taking steps to unmask in trusted spaces and beginning to explore what authenticity might look like for them, such as Peta who has “looked for more opportunities to I guess unmask or show my weaknesses around people that I trust.”

#### Subtheme 3.2: “Just made you be kinder to yourself.”

Following their diagnosis, participants described a shift toward self-compassion and self-acceptance, focusing on recognizing and respecting their own needs. As Sally expressed, she began “trying to be kinder to myself.” This emerging sense of self-acceptance often involved adjusting long-held expectations to better align with their authentic selves. As Mika noted, they treated themselves with “a lot more self-compassion since the diagnosis.” Sam reflected on the realization of their prior neglect, “I just realized how much I wasn’t looking after my health, like I was prioritizing responding to how other people wanted from me without like ever pushing back very much.” Many participants highlighted the positive effects of self-acceptance not only on their own lives but also on their relationships with others, such as their autistic children, “it’s part of who I am. And the more I accept that, the more I’m hoping my children accept that, which is the biggest reason I’m so positive about talking about this” (Bonnie).

### Theme 4: navigating self-advocacy and educating others

The final theme describes the complex interplay between participants’ emerging drive for self-advocacy and the challenges they face in educating others about autism. This theme reveals how newly diagnosed autistic adults navigate their evolving role as both learners and educators in their various life contexts. The motivation for advocacy often extended beyond personal benefit, as Bonnie explained, “the advocating one because I’m such a big thing for not only . . . my own (Autistic) children, but just changing the way people see and look at who we are.” While most participants spoke broadly about their advocacy efforts, Katie explicitly mentioned using the “Welcome Pack” to support her self-advocacy and help others understand autism:

I actually think this has good information for the (therapy) clients that we work with. Or, you know, this might help people around me understand autism better. So, it hasn’t just been like, this is my little book for me, but I’ve sort of been sharing it with people.

Other participants demonstrated creativity in how they shared autism-related information, such as making “a PowerPoint to share with people, so I didn’t have to talk to them” (Liz). However, this newfound role as an educator often presents significant challenges, particularly in professional contexts. Robyn articulated this struggle clearly:

My workplace isn’t set up to deal with my disability, and so many people have so little knowledge, and yeah, it’s almost like there’s this new burden that maybe can help with some of the others, but I can’t really. I feel the struggle of not knowing where to even start with that.

The challenge extends to family settings, such as how generational differences can play a significant role:

Educating family members continues to be challenging, given the generational knowledge about autism, there are many misconceptions and preconceptions about it—it starts very much as skepticism or dismissal, and then grows progressively once they realize that they themselves may be Autistic. (Tyler)

## Discussion

Our study explores the qualitative experiences of newly diagnosed autistic adults engaging with a neurodiversity-affirming “Welcome Pack” as they navigate their early post-diagnosis journey. Our sample was self-selecting, consisting of participants who actively chose to engage with the “Welcome Pack” and share their experiences. This is important to acknowledge, as those who participated may have been particularly motivated to seek post-diagnostic resources and reflect on their experiences in depth. Within this context, the findings provide valuable insights into how accessible and affirming resources may help to validate identity, foster a sense of community, and promote self-compassion and acceptance during a critical period of self-discovery.

Participants frequently described feeling less isolated after receiving the “Welcome Pack.” They attributed this, in part, to the inclusion of diverse and relatable biographical narratives, which helped them feel connected to a broader autistic community. Many participants reported that reading about the experiences of others who had faced similar challenges helped them feel seen and understood, potentially reducing feelings of alienation. This reflection aligns with existing research emphasizing the importance of fostering community and belonging during the early post-diagnostic period (Cage et al., 2024; Crompton et al., 2022; Norris et al., 2024). For many, this sense of solidarity appeared to support their process of embracing an autistic identity, highlighting the value of resources that emphasize shared experiences and diverse representations of autistic lives.

These findings resonate with evaluations of group-based programs, such as *Exploring Being Autistic* (Crane et al., 2020, 2023) and other structured post-diagnostic initiatives (Davies et al., 2024; Hatton & Lee, 2022), which have demonstrated the benefits of creating spaces where newly diagnosed individuals can connect with others who share their experiences. Both group-based and self-guided resources like the “Welcome Pack” appear to fulfill a critical role in reducing isolation and fostering a sense of belonging. However, while group-based programs often rely on facilitated discussions and interpersonal interactions to build community, the “Welcome Pack” supports this through self-directed engagement with narratives and reflections.

In addition to fostering connection and validation, participants reflected on the emotional complexity of their post-diagnostic journey—describing a duality of grief over years of masking and the relief of self-discovery. These reflections echo findings from other studies exploring the psychosocial complexities of late autism diagnoses (de Broize et al., 2022; Gellini & Marczak, 2023; Kelly et al., 2024; Kiehl et al., 2024; Leedham et al., 2020; Wilson et al., 2023). For some participants, engaging with the “Welcome Pack” appeared to offer an opportunity to process these emotions and begin cultivating self-compassion. It also seemed to support participants in understanding the concept of masking and its impact on their interactions and sense of self, contributing to a desire to explore what living more authentically might mean for them. These are gradual and individualized processes, but they hold significant potential, particularly given evidence that autistic adults often report lower levels of self-compassion (Cai et al., 2023) and that masking is associated with serious mental health challenges (Cook et al., 2021).

However, participants also highlighted unmet needs for practical guidance in navigating specific challenges, such as workplace advocacy, unmasking strategies, and communicating their diagnosis/identity to others. While these areas fall outside the intended scope of the “Welcome Pack,” which focuses on fostering a sense of belonging and welcoming individuals into the autistic community, the findings highlight the need for complementary resources. For example, tools such as disclosure guides (Han et al., 2024; Love et al., 2023) could provide targeted, actionable advice to newly diagnosed adults, addressing the practical challenges that many participants described.

### Implications for practice

This study emphasizes the need for comprehensive and neurodiversity-affirming post-diagnostic support systems for newly diagnosed autistic adults. Diagnosticians and service providers can play a pivotal role in bridging the gap between diagnosis and meaningful support by actively promoting resources like the “Welcome Pack.” By ensuring that such tools are introduced early and paired with opportunities for community connection, service providers can alleviate some of the uncertainty and isolation that often follow a diagnosis. Autism and disability organizations can further amplify the impact of these resources by extending their reach to those who may not yet be engaged with formal diagnostic or support systems. This proactive approach is particularly important given the significant barriers adults often face when seeking a formal diagnosis (Ardeleanu et al., 2024) and the likelihood that adults have already engaged with a substantial amount of reading and self-reflection before receiving their diagnosis (de Broize et al., 2022).

However, no single resource can address the full spectrum of support needs. Self-guided resources like the “Welcome Pack” provide an accessible and scalable way to offer validation, foster connection, and support identity exploration. However, such tools cannot and should not be considered a substitute for peer support or interpersonal interactions facilitated by group-based programs (Mandy, 2022). Instead, self-guided resources and group-based programs should be seen as complementary elements of a more connected and holistic post-diagnostic ecosystem. While self-guided tools can reduce accessibility barriers such as cost and location, group-based programs provide opportunities for direct peer interaction, reciprocal relationships, and facilitated community-building, which many autistic adults value.

The stakes are high: without coordinated and accessible supports, autistic adults risk navigating an often pathologizing information landscape, which can hinder self-acceptance and exacerbate challenges in developing a positive autistic identity (Beresford & Mukherjee, 2025; Skafle et al., 2024). While service providers face resourcing constraints (Crane et al., 2018; Crompton et al., 2022), systemic investment in post-diagnostic supports is not just an ethical imperative—it is essential for fostering societal inclusion and improving the well-being of autistic adults. Policymakers and funding bodies must prioritize equitable allocation of resources for these supports, recognizing them as integral to diagnostic pathways (Nayyar et al., 2025). Without such investment, service providers will remain constrained in their ability to meet the critical needs of autistic adults effectively.

### Limitations

Our study has several limitations that must be considered. The sample, while rich in depth, was relatively small and predominantly comprised White, female-identifying participants. The perspectives of individuals from underrepresented or multiply marginalized backgrounds, including those from culturally and linguistically diverse communities, remain underexplored. In addition, the timing of data collection may have influenced participants’ responses. Conducting interviews within 6–8 months of diagnosis provided valuable insights into early post-diagnostic experiences; however, this approach may have excluded long-term reflections on how support needs evolve over time. The study also focused specifically on experiences tied to formal autism diagnoses. While this provided a clear framework for participant recruitment, it excludes the perspectives of self-identified autistic individuals, who often face similar challenges yet remain underrepresented in research (Ardeleanu et al., 2024). Furthermore, while the “Welcome Pack” was situated as part of a broader ecosystem of post-diagnostic supports, this study did not fully explore its interaction with other resources or supports, leaving questions about its complementary role unanswered. In addition, the “Welcome Pack” is a text-based resource, which may limit accessibility for autistic adults who experience reading difficulties, including those with dyslexia or other co-occurring conditions that impact literacy.

### Future research

Future research should address these limitations by engaging larger and more diverse samples to better capture the breadth of autistic adults’ experiences, including those from multiply marginalized backgrounds. Longitudinal studies are needed to examine how the needs of newly diagnosed individuals evolve over time and how resources like the “Welcome Pack” may impact long-term mental health outcomes. Comparative studies exploring the efficacy of self-guided versus group-facilitated formats of resources would provide valuable insights into tailoring post-diagnostic supports. In addition, investigating how self-guided tools integrate with other forms of support, such as peer-led programs or facilitated interventions, could offer a more comprehensive understanding of their role in promoting well-being. Future adaptations could explore alternative formats, such as audio- or visual-based content, to enhance accessibility for autistic adults who experience reading difficulties. Codesigned studies that actively involve autistic individuals at every stage remain essential, ensuring future research and resources center lived experience and reflect the diverse realities of autistic adults.

## Conclusion

Our study highlights the importance of neurodiversity-affirming resources, such as the “Welcome Pack,” in addressing gaps in post-diagnostic support for newly diagnosed autistic adults. By offering validation, fostering a sense of belonging, and promoting self-compassion, the “Welcome Pack” provides a valuable foundation for individuals navigating the complexities of their autistic identity. Although this study was designed to explore participants’ experiences with the “Welcome Pack” rather than to evaluate its effectiveness as an intervention or to assess broader post-diagnostic services, our findings underscore that self-guided resources can play a pivotal role but must be complemented by more sustained and personalized supports, including peer connections and practical guidance tailored to individual needs. To ensure holistic care, greater investment in post-diagnostic services that integrate resources like the “Welcome Pack” with broader community-based and systemic supports is essential. By bridging these gaps, post-diagnostic care can empower autistic adults to thrive in their authenticity and foster greater societal understanding of autism.

## Supplemental Material

sj-docx-1-aut-10.1177_13623613251335070 – Supplemental material for “I’m not feeling alone in my experiences”: How newly diagnosed autistic adults engage with a neurodiversity-affirming “Welcome Pack” Supplemental material, sj-docx-1-aut-10.1177_13623613251335070 for “I’m not feeling alone in my experiences”: How newly diagnosed autistic adults engage with a neurodiversity-affirming “Welcome Pack” by Chris Edwards, Abigail MA Love, Ru Ying Cai, Melanie Heyworth, Alexandra Johnston, Fiona Aldridge and Vicki Gibbs in Autism
