# Supplementary material for: “I’m not feeling alone in my experiences”: How newly diagnosed autistic adults engage with a neurodiversity-affirming “Welcome Pack”
Source: Autism. 2025 Apr 25;29(8):2072–83. doi: 10.1177/13623613251335070 (PMC12255835; doi:10.1177/13623613251335070)
Supplement: sj-docx-1-aut-10.1177_13623613251335070 – Supplemental material for “I’m not feeling alone in my experiences”: How newly diagnosed autistic adults engage with a neurodiversity-affirming “Welcome Pack” [file sj-docx-1-aut-10.1177_13623613251335070.docx]

Thank you for taking the time to participate in this interview. We have a range of questions prepared, but please don't feel that you need to answer all of them. Some questions might feel repetitive, and that's okay—we're aiming to gather a comprehensive understanding of your experiences.

There are two key areas we're particularly interested in:

1. Your experiences following your autism diagnosis, particularly after having the ‘Welcome Pack’ for about two months.
2. How the ‘Welcome Pack’ could be improved to better support newly diagnosed Autistic adults.

As we go through the questions, please share whatever you feel comfortable with, and remember there's no right or wrong answer. Your insights are valuable in helping us understand and improve the resources available to newly diagnosed Autistic adults.

1. Since your autism diagnosis, we'd love to know how you've been doing. Have you noticed any shifts in the way you see yourself or go about your daily activities? Can you share some examples?
2. Can you describe the type of support or information you were looking for after receiving your autism diagnosis?
3. How did you feel about the 'Welcome Pack' when you first looked through it? Were there any parts that stood out or were especially meaningful to you?
4. How has the 'Welcome Pack' been a part of your journey since receiving your autism diagnosis?
5. Did the 'Welcome Pack' present views on autism that felt right to you? How did these views match up with your own feelings or thoughts about being Autistic?
6. How, if at all, did the 'Welcome Pack' influence your views on:
   - Your sense of being Autistic?
   - Being kind to yourself?
7. Did the 'Welcome Pack' prompt you to think about camouflaging or masking? Can you share how it influenced your thoughts on these topics?
8. How was your experience interacting with the 'Welcome Pack'? Did anything in particular make it easier or harder for you to engage with it?
9. What are your thoughts on how clear and accessible the content and layout of the 'Welcome Pack' were?
10. How did you feel about the way the worksheets and resources were presented? Did you find them useful?
11. From the list of topics (share screen – Parts 1-7) included in the 'Welcome Pack,' which did you find particularly useful or relevant? Why?
12. Did any parts of the 'Welcome Pack' not really connect with you or engage you as much? Could you share which parts and why?
13. Do you believe any topics require more depth or were there topics you felt were missing?
14. Do you have any ideas or suggestions for how it might be improved?
15. Is there anything else you would like to share about the ‘Welcome Pack’?
